# Striatal Dopaminergic Deficit and Sleep in Idiopathic Rapid Eye Movement Behaviour Disorder: An Explorative Study

**DOI:** 10.2147/NSS.S267037

**Published:** 2021-01-06

**Authors:** Danielle Wasserman, Dorothea Bindman, Alexander D Nesbitt, Diana Cash, Milan Milosevic, Paul T Francis, K Ray Chaudhuri, Guy D Leschziner, Luigi Ferini-Strambi, Clive Ballard, Amy Eccles, Ivana Rosenzweig

**Affiliations:** 1 Sleep and Brain Plasticity Centre, Department of Neuroimaging, Institute of Psychiatry, Psychology and Neuroscience (IoPPN), King’s College London, London, UK; 2 Sleep Disorders Centre, Guy’s and St Thomas’ NHS Foundation Trust, London, UK; 3 Headache Group, Department of Clinical Neurosciences, King’s College Hospital NHS Foundation Trust, London, UK; 4 BRAIN, Department of Neuroimaging, King's College London, London, UK; 5 School of Public Health “Andrija Stampar“, University of Zagreb School of Medicine, Zagreb, Croatia; 6 Wolfson Centre for Age-Related Diseases, King’s College London, London, UK; 7 Movement Disorders Unit, King’s College Hospital, Department of Clinical and Basic Neurosciences, Institute of Psychiatry, Psychology & Neuroscience, Parkinson Foundation Centre of Excellence, King’s College London, London, UK; 8 Sleep Disorders Center, Department of Clinical Neurosciences, Università Vita-Salute San Raffaele, Milan, Italy; 9 Medical School, University of Exeter, Exeter, UK; 10 Department of Nuclear Medicine, Guy’s and St. Thomas’ NHS Foundation Trust, London, UK

**Keywords:** isolated rapid eye movement behaviour disorder, polysomnography, sleep architecture, striatum, striatal dopamine transporter uptake tracer signalling imaging, DaTSCAN

## Abstract

**Introduction:**

Idiopathic rapid eye movement (REM) sleep behavior disorder (iRBD) is increasingly recognised as an important precursor disease state of alpha-synucleinopathies. This parasomnia is characterized by a history of recurrent nocturnal dream enactment behaviour, loss of skeletal muscle atonia, and increased phasic muscle activity during REM sleep. Neuroimaging studies of striatal dopamine transporter uptake tracer signaling suggest increasing dopaminergic deficit across the continuum of the alpha-synucleinopathies, with early sleep dysfunction suggestive of early caudate dysfunction. Henceforth, we set out to investigate the relationship between early sleep changes and the striatal dopaminergic availability in iRBD.

**Methods:**

Twelve patients with iRBD, who had undergone a video polysomnography and a neuroimaging assessment of striatal dopamine transporter (DaT) uptake tracer signaling, and 22 matched controls who had similarly undergone a video polysomnography were retrospectively identified. Data were statistically analyzed to identify altered sleep parameters and correlate them with striatal dopamine transporter uptake tracer signaling.

**Results:**

The iRBD patients exhibited an increased number of periodic limb movements during sleep (*P*=0.001), compared to 22 age-matched healthy subjects. In addition, several significant links were found between regional DaT-uptakes and sleep architecture. Correlational analyses suggested a strong positive association between sleep fragmentation and dopamine deficiency in left caudate (r=−0.630, *P*=0.028), whilst an increased uptake in the whole striatum was strongly linked to the sleep efficiency, and to a lesser degree to the length of sleep duration.

**Discussion:**

To the best of our knowledge, this is the first demonstration of a close relationship between dopaminergic availability in striatum and the quality of sleep in iRBD. Taken together, our exploratory findings suggest that subtle but functionally significant striatal changes in early stages of iRBD may contribute to the further shaping of sleep architecture.

## Introduction

Idiopathic rapid eye movement (REM) sleep behaviour disorder (iRBD) was originally described as a rare, dream re-enacting primary parasomnia.^1^ It is characterised by the absence of REM-related muscle atonia, accompanied by jerks and motor behaviours reflecting REM-related mentation.^2^ Later recognition of iRBD as the prodromal stage of an α-synucleinopathy, such as Parkinson’s disease (PD), dementia with Lewy bodies or multiple system atrophy, suggests that clinically isolated iRBD may present a unique opportunity to define novel biomarkers, to help elucidate recognizable precursory neurodegenerative states, and to identify treatment targets.^3^,^4^ Amongst known biomarkers, reduced striatal-dopamine-transporter-uptake (DaT) tracer-signalling imaging has been identified as an important tool to help define proximity to conversion to overt α-synucleinopathy in a series of pivotal studies.^5–7^ In addition, gradually increasing loss of DaT-uptake has been demonstrated across the continuum of isolated RBD to RBD and PD.^3^

Over the past few decades, neuroimaging studies of PD have consistently demonstrated uneven dopaminergic deficit within the striatum, with more severe involvement of the posterior putamen and a relative sparing of the head of caudate nucleus.^3^,^8^ This asymmetrical posterior-to-anterior gradient of dysfunction does not appear to change substantially with disease progression.^8^ More recently, however, the occurrence of early caudate dysfunction has been proposed to confer higher burden of non-motor comorbidities, such as depression and cognitive impairment, with overall worse prognosis.^9^,^10^ In keeping with this, some authors have hypothesised that earlier onset of RBD or PD in a course of neurodegeneration may depend on whether the dorsal or ventral part of the brainstem are initially involved.^10^ Accordingly, sleep issues in the disease process are more likely to appear first if the lesions start in the caudoventral mesopontine junction where caudate-labelled cells predominate.^10^

To date, surprisingly little is still known about macroscopic and microscopic sleep structure in iRBD,^4^,^11–13^ and even less so about its relationship with dopaminergic deficit within the striatum. The aim of this exploratory study was hence to investigate the relationship between early sleep changes and striatal functionality in iRBD in terms of both the severity and topography of nigrostriatal deafferentation by means of ^123^I-ioflupane-SPECT data in *de novo* iRBD patients, as well as in comparison with polysomnographic (PSG) data from healthy controls.

## Materials and Methods

**Figure 1:**
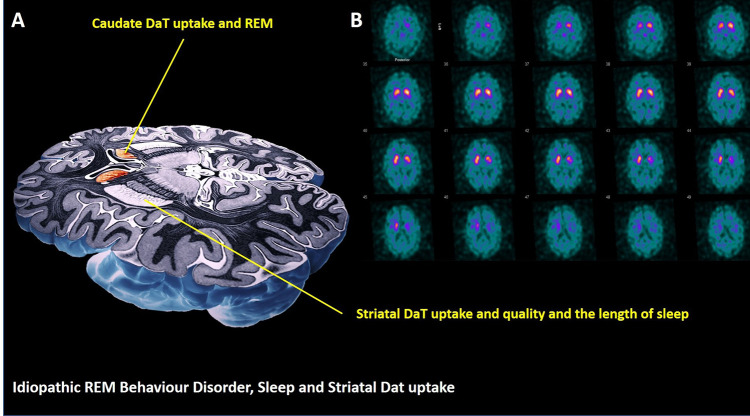
Schematic presentation (**A**) of links between sleep in iRBD and caudate and putamen DaT uptakes. Serial SPECT images from representative iRBD patient (**B**) show labelling of the basal ganglia with ^123^I-FP-CIT.

A retrospective analysis of clinical and PSG findings of patients diagnosed with iRBD based on the American Academy of Sleep Medicine (AASM) classification,^14^ who had undergone at least one single photon emission computerized tomography (SPECT) scan with ^123^I-ioflupane at a large tertiary sleep centre between 2009 and 2019 was conducted (Table 1, Figure 1A). The study was granted an ethical approval by the Hospital Clinic Research Ethics Committee (Project-No-9585, GSTT NHS, UK), which did not require informed patients’ consent for retrospectively ascertained anonymized data where the study protocol was judged to abide by the strictest patients’ data confidentiality and when it complied with the EU General Data Protection Regulation and with the Declaration of Helsinki. Of fifteen eligible records, three were excluded due to incomplete data (n=2) and a previous diagnosis of PD (n=1). Twelve patients with no neurologic comorbidity were eventually identified, and their eligibility confirmed through clinical records and the sleep diaries.

**Table 1:** Sociodemographic and Polysomnography Data of iRBD Cases (n=12) and Controls (n=22)

			iRBD N (%)	Controls N (%)	*P* value (Fisher’s Exact Test)		
Gender		Male	11 (91.7)	20 (90.9)	1.000		
		Female	1 (8.3)	2 (9.1)			
Medication: antidepressant		No	9 (75)	19 (86.4)	641		
		Yes	3 (25)	3 (13.6)			
		**Minimum**	**Maximum**	**25th**	**50th (Median)**	**75th**	***P* value (2-Tailed; Mann–Whitney U)**
Age (years)	iRBD	57.00	79.00	62.25	66.50	69.75	0.732
	Controls	54.00	82.00	60.75	66.00	69.25	
TST	iRBD	243.00	475.50	251.75	348.00	400.25	0.262
	Controls	147.00	443.50	208.25	292.00	374.75	
WASO (min)	iRBD	27.50	188.40	67.95	101.90	144.03	0.465
	Controls	39.30	230.60	74.95	117.00	169.10	
SL (min)	iRBD	9.00	95.50	18.33	33.00	47.38	0.056
	Controls	1.00	166.00	8.25	18.60	30.75	
SE (%)	iRBD	52.00	94.80	55.70	75.60	84.84	0.231
	Controls	37.10	88.70	52.25	65.90	78.60	
REM Latency (min)	iRBD	50.00	228.00	60.50	83.00	212.00	0.840
	Controls	10.00	345.00	65.25	86.25	136.63	
AHI (events/hr)	iRBD	0.00	25.80	0.50	2.80	11.43	0.759
	Controls	0.00	28.50	1.40	2.85	6.88	
PLMI (events/hr)	iRBD	1.20	139.10	11.50	28.30	56.50	0.001*
	Controls	0.00	49.00	0.00	3.15	11.18	
%N1	iRBD	3.40	38.40	12.03	13.15	21.58	477
	Controls	4.40	59.20	9.95	16.00	23.80	
%N2	iRBD	14.30	48.90	28.55	39.60	47.23	866
	Controls	25.20	82.00	30.10	38.60	50.75	
%N3	iRBD	15.20	49.90	18.28	22.35	36.78	477
	Controls	13.60	54.90	17.80	21.00	27.20	
%REM	iRBD	0.00	31.70	14.00	20.55	26.70	107
	Controls	0.00	31.40	4.50	14.80	20.10	

**Note:** *(in bold), denotes statistically significant results (P< 0.05).

**Abbreviations:** %, percentage; AHI, apnoea/hypopnoea index; N, number; N1-3, non-REM sleep stages one to three; NREM, non-rapid eye movement sleep; PLMI, periodic limb movement index; REM, rapid eye movement; SE, sleep efficiency; SL, sleep latency; TST, total sleep time; WASO, wakefulness after sleep onset.

Once the iRBD cohort was defined, simple demographics (age, sex) were used to select matched control subjects from a subset of patients entered into the same database, over the same period, with unremarkable PSG (Table 1). Control subjects presented with non-diagnostic sleep complaints; the search term used to initially mine the database was “unrefreshing sleep” entered as the patient’s presenting complaint. Moreover, all control subjects had to have unremarkable pre-admission sleep diaries and overnight video PSG studies. For the purposes of this study, unremarkable was defined as evidence of normal NREM-REM sleep cycling, with no evidence of sleep pathologies including sleep-disordered breathing, sleep-related movement disorders, NREM or REM parasomnias or wake-sleep-REM transitional instability.^15^ Twenty-two eligible records were identified in the database that satisfied that inclusion and exclusion criteria, and were subsequently further reviewed by the independent clinician to ensure that no overt neurological or general medical comorbidities existed.^15^

### Polysomnography and SPECT Data Acquisition

All subjects had undergone overnight PSG, and the subsequent sleep scoring and video-analysis were performed in accordance with AASM by two experienced sleep technologists.^14^ Subsequently, pre-agreed general measures of sleep efficiency were collected, such as total-sleep-time (TST), wake-time-after-sleep-onset (WASO) and sleep latency (SL). Further measures derived from polysomnography that reflect quality and architecture of sleep, included REM latency (e.g., REM L), percentage of total sleep time in NREM stages one to three and REM sleep (e.g., %N1, %N2, %N3, %REM), and those that likely reflected sleep fragmentation, such as periodic limb movement index (PLMI) and apnoea/hypopnoea indices (AHI), were collected.^16^

SPECT imaging was done following an overnight PSG, and the SPECT images were acquired 3-hours after intravenous bolus injection of ^123^I-ioflupane, as previously described.^9^ Regions of interest (ROIs) were placed over the right and left putamen, caudate and occipital cortices in each subject.^17^ Tomographic imaging data were reconstructed using BRASS (brain reorientation and analysis, HERMES Medical Solutions, Sweden), as previously described.^17^

### Data Analyses

Statistical interrogations were performed using the Statistical Package for the Social Sciences V.21 (IBM SPSS Statistics for Windows, version 25.0). Data were assessed for normal distribution using the Kolmogorov–Smirnov test. Fisher’s exact test of independence (nominal variables) and Mann–Whitney U-test (non-parametric) were conducted; all tests used a threshold of *P* < 0.05. ^123^I-ioflupane SPECT data were analysed using the BRASS software (HERMES medical solutions, Sweden) following a semi-quantitative approach.^9^ Reconstructed images were automatically registered to a predefined template (BRASS software).^9^ A further visual and manual inspection was done where necessary, following automatic alignment to ensure fit to the predefined template.^9^ Uptake ratios of ^123^I-ioflupane were calculated for each striatum, including caudate and putamen values, relative to the non-specific uptake measured in the occipital cortex.^9^ The uptake was defined as the specific binding ratio [(region of interest (ROIs) counts–background counts)/background counts], as previously described.^9^ Specific dopamine transporter (DaT)-binding, as reflected by ^123^I-ioflupane uptake values, was calculated for both hemispheres. Similarly, the average binding for ROI was calculated per individual as the mean uptake value for both hemispheres, as previously defined.^9^

For the purposes of SPECT-analysis, images were further normalised to age and gender-matched healthy controls from the European-Database-of-DaTSCAN-of-healthy-controls (ENC-DAT)^18^ and the z-scores were calculated, reflecting the number of standard deviations (SD) from the mean for each ratio.^17^ Individual subjects were categorized as having DaT-deficit with z-scores >±2.^18^ Significant correlation between regional ^123^I-ioflupane tracer uptake and sleep parameters was tested, using Spearman’s rank correlation. Due to the low number of subjects receiving SPECT and the exploratory nature of the DaT analysis, we did not apply correction for multiple comparisons.

## Results

The patients and the controls were well matched according to age and sex, and it is notable that three patients and controls were prescribed selective serotonin reuptake inhibitors (SSRIs) antidepressants at the time of study (Table 1). Of the 12 iRBD cases included, the majority were male (91.7%). The median age at presentation was 66.50 years, ranging from 57 to 79 years (quartile-1, median, and quartile-3: 62.25, 66.50, 69.75). Over half were referred by their general practitioners (53.9%), a quarter by a general neurologist, one case was referred by an ear, nose and throat specialist and one by the local sleep clinic. The duration of major symptom of dream enacting at presentation was 39±28.4 months (mean±SD), reflecting a broad range from 6 to 96 months (median 36, mode 60). Four patients (33.3%) had one or more pre-existing neuropsychiatric diagnosis (e.g., depression, n=3; post-traumatic stress disorder, n=1), but none had overt motor or cognitive symptoms reported or elicited at presentation. Phenoconversion to alpha-synucleinopathy during the investigated period was reported for two iRBD patients; (e.g., to idiopathic PD after six years; to atypical PD after 5.4 years). No significant differences were demonstrated between iRBD group and controls in all but two of the investigated sleep parameters (Table 1). The analysis of the sleep architecture suggested higher number of periodic limb movements in iRBD group, as evident by the PLMI (*P*=0.001). A longer period of time needed to initiate sleep was similarly recorded for the iRBD group, although it did not reach statistical significance (SL: *P*=0.056).

Reduced striatal uptake, reflecting dopamine deficiency, was initially reported for nine iRBD patients (75%) following visual (unquantified) investigation; subsequent semi-automatic quantification analysis confirmed reduced DaT-uptake in eight patients (Table 2, as signified by z-scores>±2). Reduced uptake was recorded independently on the right and on the left in eight patients (66.67%), in the region of putamen. Similarly, reduced uptake was recorded independently on the right and on the left in six iRBD patients (50%) in the region of the caudate nucleus. Visual unquantified analysis led to an underestimation of reduction in DaT-uptake in the right hemisphere by comparison to the automatic quantification. The most significant divergence was noted in the visual assessment of dopamine deficiency in the region of the right caudate nuclei (e.g., 0% versus 50%).

**Table 2:** DaTSCAN Imaging Characteristics in iRBD Patients (n=12)

DaTSCAN Report Description	Visual/Unquantified Report	Semi-Quantified Analysis*	Uptake Median Values of 123I-Ioflupane SPECT by ROI
Overall result abnormal, n (%)	9 (75)	8 (66.7)	n/a
Abnormal with increased background, n (%)	7 (58.3)	n/a	n/a
Abnormal with bilateral loss of uptake in basal ganglia, n (%)	4 (33.33)	n/a	n/a
Reduction in right putamen uptake, n (%)	3 (25)	8 (66.7)	n/a
Right POR, median (z-score: quartile 1, median, and quartile 3)	n/a	n/a	1.86 (−3.43,-2.2,-1.54)
Reduction in left putamen uptake, n (%)	5 (41.6)	8 (66.7)	n/a
Left POR, median (quartile 1, median, and quartile 3 z-score)	n/a	n/a	1.91(−3.44, −2.35,-1.73)
Reduction in right caudate uptake, n (%)	0 (0)	6 (50)	n/a
Right COR, median (z-score: quartile 1, median, and quartile 3)	n/a	n/a	2.25 (−2.59, −2.02–1.27)
Reduction in left caudate uptake, n (%)	2 (15.4)	6 (50)	n/a
Left COR, median (z-score: quartile 1, median, and quartile 3)	n/a	n/a	2.37 (−3.17,-1.91,-1.15)
Reduction putamen>caudate, n (%)	5 (41.67)	n/a	n/a
Left striatum > right, n (%)	4 (33.33)	n/a	n/a
Right striatum > left, n (%)	1 (0.83)	n/a	n/a

**Note:** *Abnormal results for quantification defined as z score >±2.

**Abbreviations:** COR, caudate to occipital ratio; DaTSCAN, 123I-2β-carbomethoxy-3β-(4-iodophenyl)-N-(3-fluoropropyl)-nortropane (123I-FP-CIT) SPECT; L, left; POR, putamen to occipital ratio; R, right; ROI, regions of interest.

Further correlational analysis of links between sleep parameters and regional DaT-uptakes (Table 3) suggested a strong positive association between sleep fragmentation (e.g., as suggested by WASO) and dopamine deficiency in left caudate (L_c_: r=−0.630, *P*=0.028). Conversely, increased uptake in the whole striatum was strongly linked to the sleep efficiency (e.g., SE), and to a lesser degree also to the length of sleep duration (e.g., TST). The positive association between the dopamine availability and the period of sleep that patients spent in REM stage (REM%) was demonstrated in the left caudate (L_c_: r=0.711, *P*=0.001) and putamen region (L_p_: r=0.609, *P*=0.035). Moreover, the correlations appeared strongly driven by the presence of pathology as determined via DaT-uptake z-scores (>±2) for that region (e.g., L_c_: non-pathologic r=0.406 [n=4] versus pathologic r=0.771 [n=8; L_p_: non-pathologic r=0.316 [n=4] versus pathologic r=0.690 [n=8]). No other significant links were found between regional DaT-uptakes and sleep architecture.

**Table 3:** Correlation Between Polysomnography Derived Sleep Parameters and DaTSCAN Quantification in iRBD Patients (n=12)

Spearman Correlation		Right COR	Left COR	Right POR	Left POR
TST	r	0.622	0.546	0.723	0.585
	*P*	0.031*	0.066	0.008*	0.046*
WASO	r	−0.531	−0.630	−0.575	−0.501
	*P*	0.075	0.028*	0.051	0.097
SL	r	−0.004	−0.191	−0.209	−0.256
	*P*	0.991	0.552	0.514	0.422
SE	r	0.650	0.592	0.804	0.683
	*P*	0.022*	0.043*	0.002*	0.014*
REM L	r	−0.396	−0.365	−0.108	−0.178
	P	0.228	0.269	0.753	0.600
AHI	r	0.053	0.200	0.016	0.040
	*P*	0.871	0.533	0.961	0.901
PLMI	r	−0.175	−0.207	0.105	−0.039
	*P*	0.587	0.519	0.745	0.905
%N1	r	0.266	0.428	0.301	0.484
	*P*	0.403	0.165	0.343	0.111
%N2	r	−0.189	−0.252	−0.232	−0.287
	*P*	0.557	0.429	0.469	0.365
%N3	r	−0.119	−0.291	−0.196	−0.291
	*P*	0.713	0.359	0.540	0.359
%REM	r	0.434	0.711	0.497	0.609
	*P*	0.159	0.010*	0.100	0.035*

**Note:** *(in bold), denotes statistically significant results (P< 0.05).

**Abbreviations:** %, percentage; AHI, apnoea/hypopnoea index; COR, caudate to occipital ratio; DaTSCAN, 123I-2β-carbomethoxy-3β-(4-iodophenyl)-N-(3-fluoropropyl)-nortropane (123I-FP-CIT) SPECT; N, number; N1-3, non-REM sleep stages 1–3; NREM, non-rapid eye movement sleep; P, Spearman’s rank correlation P value; PLMI, periodic limb movement index; POR, putamen to occipital ratio; r, Spearman’s rank correlation rho value; REM, rapid eye movement; SE, sleep efficiency; SL, sleep latency; TST, total sleep time; WASO, wakefulness after sleep onset.

## Discussion

Our data converge on two key findings, that suggest a close relationship between dopaminergic availability in striatum and the quality of sleep in iRBD. Firstly, in this study, the link between sleep efficiency and sleep duration and dopaminergic availability in the striatum is demonstrated. Next, an important modulatory role for caudate in arousals, sleep fragmentation and REM mechanisms is intimated by the results (Figure 1, Table 3).

The importance of appreciation of links between dopaminergic functionality of caudate region and sleep issues, as suggested by these results, may indeed be of particular clinical note. Lately, in opposition to traditional views, nigro-caudate dopaminergic deafferentation has been argued as a principal biomarker of iRBD.^10^ Also, several recent studies have demonstrated an early involvement of caudate in patients with overall worse outcomes, and with an increased risk of developing debilitating cognitive, gait and mood problems.^8^,^10^ Moreover, unless particularly sought, the abnormalities in this region can be challenging to spot in a busy clinical setting, and an unquantified visual analysis of DaT-imaging has been shown to underestimate abnormal findings in this region of striatum, as it was also demonstrated in this study (Table 2).

Historically, unrelated to RBD disease process, both caudate nucleus and REM sleep have been linked to “orienting and alerting responses” in early animal experiments.^19^,^20^ Early studies have suggested a role for a phasic caudate-spindle rhythm^21^ in modulation of brainstem alerting signals, including REM sleep’s ponto-geniculo-occipital (PGO) waves, and in an initiation of a phasic-REM stage. Regarded as a physiological correlate of dreaming and sleep-dependant learning, PGO-waves are thought to spread through thalamocortical system leading to cortical functional synchronisation of fast (beta-band) oscillations during REM sleep.^22^,^23^ Perhaps in agreement with its altered physiological role, an altered caudate recruitment has been linked to pathologic hyperarousal, inability to initiate and maintain sleep, deficits in executive functioning,^24^ and altered timing and spatial navigation in several other disorders.^23^ In iRBD, however, previous studies have demonstrated decreased REM stage stability,^13^ along with higher beta-band increases during REM.^11^ In that background, the data in this study that link caudate dopaminergic availability and REM sleep may suggest a distinct altered caudate response in iRBD. For example, a decreased involvement of this part of nigrostriatal circuitry may initially lead to an overall plastic increase in phasic-REMs. This could then lead to increase in sleep-stage shifts and previously reported sleep-stage instability^13^ as well as altered REM mentation in iRBD.^3^

In this study, we were unable to demonstrate macrostructural sleep differences between our iRBD and control subjects. This is in some agreement with previous reports of a relatively preserved sleep architecture in iRBD patients.^11^ However, “slowing” of the sleep EEG activity has also been reported in iRBD,^4^ with the relative decrease in spindles^25^ and the relative increase in delta-band and deep sleep architecture.^11^ Moreover, overall deeper sleep of patients with RBD has been suggested in number of studies, with lower number of awakenings, lower percentage of wakefulness after sleep onset and higher sleep efficiency and a higher number of sleep stage shifts by comparison to healthy controls.^11^,^13^ In that light, the findings of correlational analyses in this study are of particular interest (Table 3). Strikingly, a strong association was demonstrated between the pathologic DaT-scores in the left putamen (and caudate) and sleep efficiency, total sleep time and percentage of time spent in REM sleep. In addition, an inverse relationship with WASO scores was also shown. Arguably, this suggests that profound plastic changes, and an aberrant striatal neurocircuitry, may be already present at this early stage of disease process (Table 3). Whilst still speculative, several preclinical studies of early biomarkers in PD appear to support this notion. For instance, in one such study, lesion in pedunculopontine tegmental nucleus led to an early reorganizational modulation of basal ganglia that included striking early loss of striatal cholinergic interneurons and striatal hypersensitivity to dopamine associated with early microscopic and macroscopic sleep changes.^26^ Of note is that, in a recent large multicentre study that combined prospective follow-up data from 24 centres of the International RBD Study Group, the rate of phenoconversion was reported to be significantly increased with abnormal DaT-scores.^27^ Here, the dopaminergic availability in the putamen was pre-chosen as a sole region of interest, somewhat limiting targeted comparisons with our study. Further alternative explanations of the findings in our study, however, might be also explained by a permissive nature of wider brain pathology, including previously proposed breakdown in REM-on sleep circuitry due to ongoing neurodegeneration in the region of the sublaterodorsal tegmental nucleus.^11^,^28^

Finally, in this exploratory study, iRBD patients were recorded with a higher number of PLMs than control subjects. This result is in keeping with previous reports and may be reflective of shared basal ganglia etiology, which we were unable to demonstrate in this study.^29^,^30^ It is likely that demonstration of any such shared etiology will in future require a concomitant prospective spatio-temporal examination of changes in striatal circuitry during course of iRBD. Of note, in iRBD, reduction of cardiac and EEG activation associated with PLMS has also been demonstrated, suggesting the presence of an impaired autonomic and cortical reactivity to internal stimuli.^29^ Given the previously hypothesised plastic changes in nigrostriatal “sensing”-systems, it is tempting to suggest that our proposed aberrant caudate activation in iRBD might also underlie some of these findings. Similarly, it might also contribute to increased sleep latency recorded in iRBD patients.

Lastly, it should be noted that depression and mood changes have been increasingly recognised amongst the important prodromal non-motor symptoms of an α-synucleinopathy process, albeit their specificity and the exact underlying mechanisms remain elusive.^31^,^32^ In keeping with this notion, four of our iRBD patients had recorded mood changes and an established psychiatric diagnosis of depression and PTSD (one patient), whilst three were treated with antidepressants at time of the investigation. The link between antidepressants and development of RBD has been historically well documented, and more recently it has been argued that rather than being just an unpleasant side-effect of an antidepressant treatment, development of iRBD presents an early signal of an underlying neurodegenerative disease.^33^ Correspondingly, patients with PTSD have also been shown to have an elevated risk of developing PD in later life.^31^,^34^ More recently, the degeneration of the locus coeruleus (LC), the major noradrenergic nucleus in the brain and a part of the central arousal system, has been argued to underlie associated affective symptoms in iRBD.^35–38^ In that context, it is of note that LC’s ascending inputs to nuclei within striatum are known to have an inverse effect, perhaps suggesting that any degenerative process in that region may similarly trigger diverse and complex changes in the wider circuitry. For example, LC has been shown to play the role in entraining the putamen and pallidum in rapid actions to external stimuli, likely in preparation for effortful cognitive actions.^39^ Conversely, its ascending inputs act to suppress immediate caudate response in favour of planned actions.^39^

In summary, to best of our knowledge, this is the first study that has linked early changes in sleep quality in iRBD with dopaminergic availability in the striatum. However, there are several important limiting factors to this exploratory study (e.g., retrospective observational nature, small size, intake of antidepressants in three patients, the lack of DaT-imaging in controls), which will need to be rectified in future larger, prospective multimodal neuroimaging studies of iRBD, in order to truly authoritatively address the causality and directionality of implied associations. Nonetheless, we believe that the findings presented here raise some fundamental questions. They also suggest subtle but functionally significant alterations in the striatal region of brains of patients with iRBD that might represent the early expression of the supposed neurodegenerative processes already taking place at this stage of the disease, and that might be the target of better and effective future therapeutic strategies for this condition.

## Data Sharing Statement

All data that support the findings of this study are available upon request from the corresponding author.
